# Prevalence, risk factors, and zoonotic implications of gastrointestinal parasites in urban cats in Kazakhstan: A cross-sectional multicity study

**DOI:** 10.14202/vetworld.2025.1748-1758

**Published:** 2025-06-27

**Authors:** Lyudmila A. Lider, Vladimir Kiyan, Dinara M. Seitkamzina, Altay Ussenbayev, Botakoz E. Akmambaeva, Rabiga S. Uakhit, Nellya E. Mannapova, Igor Sytnik, Christian Bauer

**Affiliations:** 1 Parasitology Laboratory, Department of Veterinary Medicine, S. Seifullin Kazakh Agro-Technical Research University, 010011, Astana, Kazakhstan; 2 Laboratory of Biodiversity and Genetic Resources, National Center for Biotechnology, 010000, Astana, Kazakhstan; 3 Institute of Parasitology, Justus Liebig University Giessen, 35392, Giessen, Germany

**Keywords:** coproantigen detection, *Cystoisospora felis*, *Cystoisospora rivolta*, fecal flotation, feline endoparasites, gastrointestinal parasitism, *Giardia duodenalis*, helminths, Kazakhstan, One Health, public health risk, risk factors, stray versus owned cats, *Toxocara cati*, *Toxoplasma gondii*-like coccidia, urban cats, zoonotic parasites

## Abstract

**Background and Aim:**

Cats act as reservoirs for various gastrointestinal parasites, including species of significant zoonotic concern such as *Toxocara cati, Toxoplasma gondii*, and *Giardia intestinalis*. However, data on the prevalence and risk factors associated with feline endoparasites in Kazakhstan remain limited. This study aimed to determine the prevalence, species diversity, and risk factors of intestinal parasitic infections in urban cat populations across five major cities in Kazakhstan, thereby supporting the One Health framework for the prevention of zoonotic diseases.

**Materials and Methods:**

A cross-sectional survey was conducted from August 2023 to January 2025, involving 1,301 fecal samples collected from both client-owned and stray cats in Almaty, Astana, Oral, Qostanai, and Shymkent. Standardized Sheather’s sugar flotation was used to detect helminth eggs and coccidia oocysts in all samples, while *Giardia* coproantigen was assessed in 1,256 samples using a commercial immunochromatographic assay (FASTest® CRYPTO-GIARDIA strip test kit, MEGACOR, Austria). Prevalence differences across categories – ownership status, sex, age class, and city – were evaluated using the Chi-squared test, and odds ratios (OR) were calculated to identify significant risk factors.

**Results:**

Overall, 17.7% (230/1,301) of cats were infected with at least one intestinal parasite species. The most prevalent species were *Cystoisospora felis* (7.2%), *T. cati* (6.2%), *Cystoisospora rivolta* (2.0%), and *Giardia* (6.4%). *T. gondii*-like oocysts (*T. gondii* or *Hammondia hammondi*) were detected in 0.6% of samples. Significant variation in parasite prevalence was observed among cities. Stray cats were significantly more likely to harbor *C. felis* and *C. rivolta*. Female cats had higher odds of testing positive for *Giardia* (OR = 1.8). Infections with *T. cati*, *C. felis*, and *Giardia* showed a significant association with age, with kittens (<6 months) being approximately twice as likely to test positive for these parasites compared to adult cats.

**Conclusion:**

This study represents the first comprehensive assessment of gastrointestinal parasitism in urban cats in Kazakhstan. The detection of zoonotic parasites and identification of significant demographic risk factors underscore the need for enhanced public health strategies, including educational outreach, targeted deworming protocols, and environmental hygiene measures. Future molecular investigations are necessary to differentiate *T. gondii* from *Hammondia hammondi* and to genotype *Giardia* assemblages. Soil surveillance in public spaces is also recommended to assess environmental contamination and potential exposure risk to humans, particularly children.

## INTRODUCTION

Cats rank among the most favored companion animals globally, with an estimated population surpassing one billion individuals [1]. In Kazakhstan, the mandatory registration of companion animals, including cats and dogs, in a centralized database has been in effect since September 2023. By February 2025, official data indicated that approximately 86,850 cats had been registered, with 43,450 in Almaty and 19,750 in Astana [2]. Cats serve as hosts to a variety of endoparasitic species. Some parasites, such as *Ancylostoma tubaeforme* and *Giardia* spp., are clinically relevant in felines, while others, including *Toxocara cati* and *Toxoplasma gondii*, represent significant zoonotic threats to humans [3].

**Figure 1:**
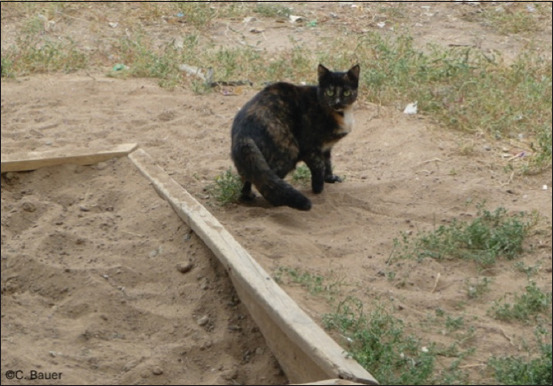
A cat defecating in a sandpit at a playground in Astana (Photo: C. Bauer).

A recent international survey spanning North America, Europe, Australia, and New Zealand revealed that around 59% of cat owners allow their pets outdoor access, with regional differences: Approximately 20% in North America versus 70% in Europe [4]. While specific data for Central Asia, including Kazakhstan, are lacking, outdoor access appears to be common. For instance, in Astana, cats are frequently seen roaming freely in residential neighborhoods (personal observations). Both client-owned and stray free-roaming cats typically defecate and bury their feces in areas such as gardens, sandpits (Figure 1), and other public environments. This behavior contributes to environmental contamination and elevates the risk of zoonotic transmission to humans, especially children, through accidental ingestion of the infective stages of *T. cati* [5] and *T. gondii* [6].

Despite the widespread ownership of domestic cats and their recognized role as reservoirs of zoonotic parasites, comprehensive data on the prevalence and distribution of gastrointestinal parasites in feline populations across Kazakhstan remain notably scarce. Existing studies are geographically limited, often focusing on specific parasites, such as *Opisthorchis felineus*, or based on small sample sizes [7–12]. Moreover, the majority of available data fail to incorporate key epidemiological variables such as host age, sex, ownership status, and geographical diversity. The lack of recent, large-scale, and methodologically standardized investigations impedes the development of effective public health policies and veterinary interventions, particularly in urban environments where close contact between humans and free-roaming cats increases the likelihood of zoonotic transmission. In the context of the One Health framework, there is a critical need for updated and geographically representative baseline data that assess not only the prevalence, but also the risk factors and zoonotic potential associated with feline gastrointestinal parasitism in Kazakhstan.

In response to this gap, the present study aimed to provide a systematic, multicity assessment of gastrointestinal parasites in urban cats across Kazakhstan. Specifically, the objectives were to (1) determine the prevalence and species diversity of intestinal helminths, coccidia, and *Giardia* spp. in both client-owned and stray cats; (2) evaluate associations between infection status and potential risk factors, including ownership status, sex, age class, and geographical location; and (3) highlight parasites of significant zoonotic concern in alignment with the One Health perspective. The study further sought to generate evidence-based recommendations to inform public health and veterinary strategies aimed at mitigating environmental contamination and reducing the risk of zoonotic transmission in densely populated urban settings.

## MATERIALS AND METHODS

### Ethical approval

This study was reviewed and approved by the Local Committee on Biological and Medical Ethics at the S. Seifullin Kazakh Agro-Technical Research University, Astana (Protocol No. 2, dated November 03, 2022). All procedures involving animals adhered to local animal welfare regulations. Permission for fecal sample collection was obtained from the directors of participating veterinary clinics and animal shelters.

### Study period and location

**Figure 2:**
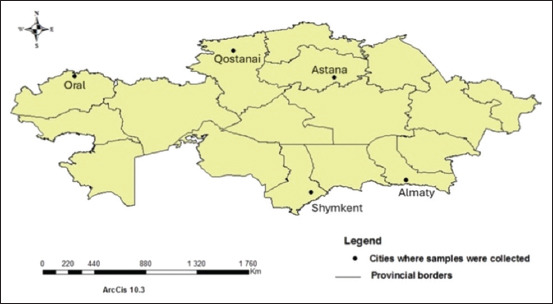
Map of Kazakhstan showing the geographical locations of the five cities where fecal samples were collected from cats [the map was generated using ArcGIS 10.3 (https://enterprise.arcgis.com)].

A cross-sectional survey was conducted from August 2023 to January 2025 in five major urban centers of Kazakhstan: Almaty, Astana, Oral, Qostanai, and Shymkent (Figure 2 and Table 1) [2, 13–15].

**Table 1:** Geographic location, climate zone, human population size, and number of registered cats in the surveyed cities of Kazakhstan.

City	Latitude and longitude^1^	Climate zone^2^	Human population size^3^	Number of registered cats^4^
Almaty	43.25°N 76.9167°E	Dfb (warm-summer humid continental climate with relatively consistent precipitation throughout the year)	2,290.000	43,450
Astana	51.1801°N 71.446°E	Dfb	1,529,000	19,750
Oral	51.2333°N 51.3667°E	Dfa (hot-summer humid continental climate)	369,000	ND
Qostanai	53.2144°N 63.6246°E	Dfb	273,000	ND
Shymkent	42.3°N 69.6°E	Csa (hot-summer Mediterranean climate)	1,125,000	ND

ND=No data are available.

1 Source: Geodatos [13].

2 Climate zones were classified according to the Köppen–Geiger classification Beck *et al*. [15].

3 Approximate number of inhabitants as of January 01, 2025, according to official statistics, Bureau of National Statistics [14].

4 As of February 2025, according to the mandatory registration database for companion animals in Kazakhstan, TAÑBA in Digits [2]

### Sample population and inclusion criteria

A total of 1,301 urban cats – comprising both client-owned and stray individuals – were sampled from 20 veterinary clinics and 10 animal shelters that voluntarily participated in the study. Eligible cats included those older than 1 month who were either presented for routine or clinical visits to veterinary clinics or admitted to shelters during the study periods. Cats with a known history of anthelmintic treatment within the preceding 4 weeks were excluded from sampling.

### Sample collection and transport

Before the initiation of sampling, study protocols were reviewed with veterinary and shelter personnel, and standardized worksheets were distributed to ensure uniformity in sample collection and data recording. Fecal samples were collected by trained staff into pre-labeled containers during veterinary consultations or upon admission to the shelter. Samples were refrigerated at 4°C–6°C and transported to the Parasitology Laboratory at S. Seifullin Kazakh Agro-Technical Research University (Astana) within 1–3 days using cold storage transport (cool bags with cold packs).

The sampling was carried out in three distinct timeframes: August 2023–February 2024, April–May 2024, and July 2024–January 2025. While sex and age data were available for most cats, information on medical history and previous antiparasitic treatments was incomplete and therefore excluded from the final analysis.

### Parasitological examination

#### Macroscopic and microscopic analysis

Each fecal sample underwent an initial macroscopic examination for the presence of cestode proglottids. Microscopic evaluation was subsequently performed using a direct Sheather’s sugar flotation method with a sugar solution of specific gravity 1.3 to detect helminth eggs and coccidian oocysts [3]. Parasite stages were identified based on morphology and size under 100× and 400× magnification [3].

#### Giardia detection

Due to sample loss or insufficient volume, 1,256 samples were available for *Giardia* testing. Detection was performed using the FASTest^®^ CRYPTO-GIARDIA strip test kit (MEGACOR, Hörbranz, Austria), following the manufacturer’s instructions. Two trained individuals independently interpreted test results. This rapid immunochromatographic assay detects cell wall antigens of *Giardia duodenalis* and *Cryptosporidium* spp. in the feces of companion animals [16]. Diagnostic validation was performed using anenzyme-linked immunosorbent assay (ELISA), which served as the reference standard, using 110 ELISA-positive and 130 ELISA-negative samples. The rapid assay showed an agreement rate of 98.3%, with a sensitivity of 97.3% and a specificity of 99.2% for *Giardia* coproantigen detection [17].

### Statistical analysis

All statistical analyses were performed using BIAS software (version 9.05; Epsilon, Hochheim, Germany) [18]. Apparent prevalences for detected parasite stages were calculated with corresponding 95% confidence intervals (CIs). Associations between parasite prevalence and categorical variables – city, ownership status, sex, and age group (1–6 months, >6 months–2 years, >2 years) – were evaluated using Chi-squared tests. Observations with incomplete data were omitted from the relevant analyses. Odds ratios (ORs) with 95% CIs were computed for the four most prevalent parasite species. Differences with p-values < 0.05 were considered statistically significant.

## RESULTS

### Study population characteristics

A total of 1,301 cats were sampled from five major urban centers in Kazakhstan: 282 from Almaty, 382 from Astana, 76 from Oral, 192 from Qostanai, and 389 from Shymkent. Among these, 39 (14%) cats from Almaty, 189 (49%) from Astana, 33 (43%) from Oral, and 128 (67%) from Qostanai were classified as strays, while all sampled cats from Shymkent were client-owned. Overall, 912 cats (70.1%) were client-owned and 389 (29.9%) were strays.

Of the cats for which sex was recorded, 655 (54.0%) were male and 559 (46.0%) were female. Age data were available for a subset of the population (n = 1,015). Among these, 517 cats (50.9%) were older than 2 years, 279 cats (27.5%) were between 1 and 6 months of age, and 219 cats (21.6%) were between 6 months and 2 years old.

### Parasitological findings

#### Overall prevalence

Out of the 1,301 cats examined, 230 (17.7%) tested positive for at least one gastrointestinal parasite. Parasitic stages identified included five helminths, three coccidian species, and *Giardia* spp. coproantigen (Table 2).

**Table 2:** Prevalence of coprologically detected endoparasite infections in client-owned and stray cats from five cities (Almaty, Astana, Oral, Qostanai, and Shymkent) in Kazakhstan.

Parasite	No. of cats positive/tested	Prevalence (%)	95% CI
*Toxocara cati* ^1^	81/1,301	6.2	5.0–7.7
*Capillaria* spp.^1^	5/1,301	0.4	0.1–0.9
Taeniids^1^	5/1,301	0.4	0.1–0.9
*Dipylidium* spp.^1^	1/1,301	0.08	<0.01–0.4
*Diphyllobothrium/Spirometra* spp.^1^	1/1,301	0.08	<0.01–0.4
*Cystoisospora felis* ^1^	94/1,301	7.2	5.9–8.8
*Cystoisospora rivolta* ^1^	26/1,301	2.0	1.3–2.9
*Toxoplasma gondii*-like^1,2^	8/1,301	0.6	0.27–1.2
*Giardia* ^3^	81/1,256	6.4	5.2–8.0
Co-infections			
*T. cati* + *Capillaria* spp.	1/1,301	0.08	nc
*T. cati* + *taeniids*	2/1,301	0.2	nc
*T. cati* + *C. felis*	3/1,301	0.2	nc
*T. cati* + *C. rivolta*	1/1,301	0.08	nc
*T. cati* + *Giardia*	21/1,256	1.7	nc
*T. cati* + *C. felis* + *C. rivolta*	2/1,301	0.2	nc
*T. cati* + *C. felis* + *Giardia*	7/1,256	0.6	nc
*T. cati* + *C. rivolta* + *Giardia*	1/1,256	0.08	nc
*C. felis* + *C. rivolta*	2/1,301	0.2	nc
*C. felis* + *Giardia*	10/1,256	0.8	nc

CI=Confidence interval, nc=Not calculated.

1 Fecal stages were detected using a direct modified Sheather’s sugar flotation method. ^2^Including *Toxoplasma gondii* and *Hammondia hammondi*.

3 Coproantigen was detected using rapid immunochromatographic assay

#### Parasite-specific prevalence

The most frequently detected stages were *Cystoisospora felis* oocysts (94/1,301; 7.2%) and *T. cati* eggs (81/1,301; 6.2%). *Cystoisospora rivolta* oocysts were identified in 26 samples (2.0%), while *T. gondii*-like oocysts were observed in eight samples (0.6%). Eggs of taeniids and *Capillaria* spp. were each detected in five cats (0.4%). Single samples were positive for *Dipylidium* spp. and *Diphyllobothrium/Spirometra* spp. No evidence of hookworms or *O. felineus* was found in any of the samples.

Of the 1,256 samples suitable for immunochro-mato-graphic testing, 81 (6.4%) were positive for *Giar-dia* coproantigen.

#### Co-infections

Co-infections involving two or more parasites were documented in 50 cats. The most common combinations included *T. cati* with *Giardia* (1.7%) and *C. felis* with *Giardia* (0.8%) (Table 2). These co-infections did not vary significantly by ownership status, sex, or age group.

### Geographic distribution of infections

**Figure 3:**
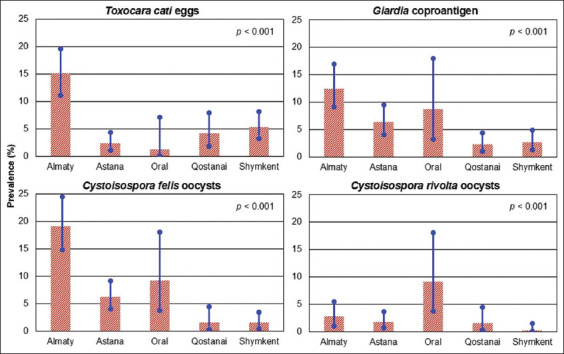
Prevalence (%) of *Toxocara cati* egg shedding, *Cystoisospora felis* and *Cystoisospora rivolta* oocyst shedding, and *Giardia* coproantigen positivity in client-owned and stray cats from five cities in Kazakhstan, with blue lines representing the respective 95% confidence intervals. p-value indicating the significance level of the differences across the cities (Chi-squared test).

The prevalence of *T. cati*, *C. felis*, and *Giardia* varied significantly (p < 0.001) across the five surveyed cities. Almaty exhibited the highest infection rates for all three parasites: 15.2% for *T. cati*, 19.1% for *C. felis*, and 12.4% for *Giardia*. In contrast, *C. rivolta* was most prevalent in oral (9.2%), diverging from trends observed in the other cities (Figure 3).

### Risk factor analysis

#### Ownership status

Statistical analysis revealed that ownership status had a significant influence on the prevalence of *Cystoisospora* infections. Stray cats were 4.7 times more likely to test positive for *C. felis* and 2.4 times more likely for *C. rivolta* compared to client-owned cats (Tables 3 and 4). No significant ownership-related difference was found for *T. cati* or *Giardia* infections.

**Table 3:** Effects of cat type, sex, and age class on the prevalence of *Cystoisospora felis* infection in urban cats in Kazakhstan.

Parameter	No. of cats positive/tested	Prevalence (%)	95% CI	p-value	Odds ratio	95% CI	p-value
Ownership status				**<0.001**			
Stray	60/389	15.4	12.0–19.4		4.7	3.1–7.1	**<0.001**
Client-owned	34/912	3.7	2.6–5.2		Reference		
Se×^1^				0.774			
Female	52/655	7.9	6.0–10.3		1.1	0.7–1.7	0.693
Male	41/559	7.3	5.3–9.8		Reference		
Age class^1^				**0.027**			
1–6 m	33/279	11.8	8.3–16.2		2.0	1.2–3.2	**0.008**
>6 m–2 y	17/219	7.8	4.6–13.1		1.2	0.7–2.3	0.497
>2 y	33/517	6.4	4.4–8.8		Reference		

CI=Confidence interval, m=Months, y=Years; p*-*values in bold=Significant.

1 Missing samples=Information not available

**Table 4:** Effects of cat type, sex, and age class on the prevalence of *Cystoisospora* *rivolta* infection in urban cats in Kazakhstan.

Parameter	No. of cats positive/tested	Prevalence (%)	95% CI	p-value	Odds ratio	95% CI	p-value
Ownership status				**0.041**			
Stray	13/389	3.3	1.8–5.6		2.4	1.1–5.1	**0.024**
Client-owned	13/912	1.4	0.8–2.4		Reference		
Se×^1^				0.521			
Female	15/655	2.3	1.3–3.7		1.4	0.6–3.3	0.396
Male	9/559	1.6	0.7–3.0		Reference		
Age class^1^				0.705			
1–6 m	6/279	2.1	0.8–4.6		0.9	0.3–2.5	0.877
>6 m–2 y	3/219	1.4	0.3–4.0		0.6	0.2–2.1	0.404
>2 y	12/517	2.3	1.2–4.0		Reference		

CI=Confidence interval, m=Months, y=Years; p*-*values in bold=Significant.

1 Missing samples=Information not available

#### Sex

Sex was significantly associated only with *Giardia* infection: Female cats were 1.8 times more likely to test positive for coproantigen than male cats (Table 5). No sex-related differences were observed for *T. cati*, *C. felis*, or *C. rivolta*.

**Table 5:** Effects of cat type, sex, and age class on the prevalence of *Giardia* infection in urban cats in Kazakhstan.

Parameter	No. of cats positive/tested	Prevalence (%)	95% CI	p-value	Odds ratio	95% CI	p-value
Ownership status				0.349			
Stray	27/354	7.6	5.1–10.9		1.4	0.8–2.2	0.187
Client-owned	54/902	6.0	4.5–7.7		Reference		
Se×^1^				**0.022**			
Female	54/637	8.5	6.4–10.9		1.8	1.1–2.9	**0.016**
Male	27/547	4.9	3.3–7.1		Reference		
Age class^1^				0.062			
1–6 m	27/272	9.9	6.6–14.1		1.9	1.1–3.3	**0.018**
>6 m to 2 y	23/311	7.4	4.7–10.9		1.4	0.8–2.5	0.248
>2 y	27/501	5.4	3.6–7.7		Reference		

CI=Confidence interval, m=Months, y=Years; p*-*values in bold=Significant.

1 Missing samples=Information not available

#### Age class

Age significantly affected the likelihood of infection with *T. cati*, *C. felis*, and *Giardia*. Kittens aged ≤6 months were nearly twice as likely to be infected with each of these parasites compared to cats older than 2 years (ORs: 1.8, 2.0, and 1.9, respectively; Tables 3, 5, and 6). *Cystoisospora* oocysts and *Giardia* coproantigen were first detected in kittens as young as 2 months, while *T. cati* eggs were primarily found in kittens older than 3 months.

**Table 6:** Effects of cat type, sex, and age class on the prevalence of *Toxocara cati* infection in urban cats in Kazakhstan.

Parameter	No. of cats positive/tested	Prevalence (%)	95% CI	p-value	Odds ratio	95% CI	p-value
Ownership status				0.495			
Stray	21/389	5.4	3.4–8.1		1.2	0.7–2.1	0.420
Client-owned	60/912	6.6	5.1–8.4		Reference		
Se×^1^				0.756			
Female	45/655	6.9	5.1–9.1		0.9	0.6–1.4	0.670
Male	35/559	6.3	4.4–8.6		Reference		
Age class^1^				0.078			
1–6 m	28/279	10.0	6.8–14.2		1.8	1.1–3.1	**0.028**
>6 m–2 y	19/219	8.7	5.3–13.2		1.5	0.8–2.8	0.153
>2 y	30/517	5.8	3.9–8.2		Reference		

CI=Confidence interval, m=Months, y=Years; p*-*values in bold=Significant.

1 Missing samples=Information not available

### Observations on rare parasites

Among the eight cats shedding *T. gondii*-like oocysts, five were strays, seven were female, and four were kittens. Of the five cats positive for taeniid eggs, three were strays, one was a kitten, and four were adults. *Capillaria* eggs were exclusively observed in client-owned cats older than 2 years. A single instance of *Diphyllobothrium/Spirometra*-like egg shedding was documented in a stray kitten.

## DISCUSSION

This study represents the first large-scale investigation of gastrointestinal endoparasites in urban cats in Kazakhstan. Nine distinct parasite taxa were identified, including *T. cati*, *T. gondii*-like coccidia, and *Giardia* spp. The study was geographically restricted to five cities, and the sampling was not proportional to the total cat population in each city. Fecal samples were obtained from veterinary clinics and animal shelters that voluntarily participated. This sampling approach constitutes a “non-probability” design [19]; therefore, the findings may not be statistically generalizable to the entire feline population in Kazakhstan. Moreover, no specific information was available regarding the individual cats examined, including deworming frequency, diet, or housing conditions. Nevertheless, the data provide valuable insights into the prevalence of parasites, their geographic distribution, and the associated risk factors for endoparasites within urban areas of Kazakhstan. These findings provide essential baseline information for municipal administrations, aligning with the principles of the One Health approach, to support efforts in controlling zoonotic parasites transmitted by cats. Recommended measures include enhancing and expanding the education of pet owners about infection risks and prevention strategies (public awareness campaigns), monitoring public areas for the presence of parasite stages in soil, replacing sand in public sandpits, and intensifying efforts to manage and reduce stray cat populations.

### T. cati

*T. cati* emerged as the predominant gastrointesti-nal nematode, consistent with previous reports [20–25]. The overall prevalence of egg shedding was relatively low at 6.2% across the sampled cities. This percentage is comparable to reports from Moscow (4.1% [26]) and nationwide surveys in Germany (3.5% [25]) and the USA (4.6%–5.1% [22]) but significantly lower than in many other countries, where the pooled global prevalence is estimated at 17%, including 24.3% in urban cats [27, 28]. Notably, no significant difference in *T. cati* egg shedding was observed between stray and client-owned cats (Table 6). This outcome contrasts with previous studies [20, 27, 28] and may indicate either similar exposure risks among both urban client-owned and stray cats or suboptimal deworming practices in client-owned animals in Kazakh cities.

Consistent with a prior investigation by Rostami *et al*. [27], the frequency of *T. cati* egg shedding did not differ significantly between males and females. *T. cati* eggs were not detected before the end of the 2^nd^ month of life. This reflects the parasite’s biological characteristics: *T. cati* does not undergo prenatal (transplacental) transmission, in contrast to *T. canis*, which exhibits transplacental transmission in dogs [3]. Cats become infected with *T. cati* only postpartum by ingesting embryonated eggs from the environment or larvae in paratenic hosts (prey) or through the lactogenic route through the milk of recently infected queens [29–31]. Consequently, they begin shedding eggs after a prepatent period of approximately 8 weeks [29]. Egg shedding occurred significantly more frequently in kittens than in adults, in agreement with earlier findings [20, 27]. This may be due to kittens becoming infected after birth, whereas the lower prevalence in older cats indicates acquired partial immunity to this roundworm species.

*T. cati* prevalence differed significantly across the surveyed cities. In Almaty and Astana, for example, the rates were 15.2% and 2.4%, respectively (Figure 3). The reasons for these local differences remain unclear; however, it is uncertain whether climatic or biological factors, for example, play a role. Assuming these results are tentatively representative of the local cat populations and considering the number of registered animals, such as the 43,450 cats in Almaty [2], a speculative extrapolation suggests that at least 6,700 cats in this city were shedding *T. cati* eggs at the time of the study. Similar to dogs infected with T. canis, free-roaming cats shedding *T. cati* eggs present a tangible infection risk to humans, particularly children [32]. It would therefore be appropriate to test soil in public parks and playground sand in urban areas for roundworm eggs using suitable diagnostic methods to assess the extent of environmental contamination [33, 34].

The seroprevalence of *Toxocara* antibodies in the human population can vary greatly across different cities and regions within a country, as observed in Russia (5%–40% in children [35]). In Kazakhstan, data on this topic remain fragmentary, in contrast to echinococcosis and opisthorchosis [36–38]. However, a seroprevalence of *Toxocara* antibodies of 11% has been reported in a rural population in southeastern Kazakhstan [39]. Consequently, toxocarosis should be recognized as a neglected zoonotic disease in Kazakhstan.

*T. cati* is pathogenic not only to its definitive feline host, where it can cause significant lung disease with pulmonary arterial, bronchial, and interstitial alterations [40, 41], but also to paratenic hosts. In mice and pigs (paratenic hosts) experimentally infected with *T. cati*, larvae migrate into various tissues, including the brain, leading to pathomorphological alterations and, in mice, abnormal neurobehavior [42, 43]. Therefore, similar to *T. canis* in dogs, *T. cati* should also be recognized as a potential cause of clinical symptoms in humans [5]. Depending on the number of *Toxocara* eggs ingested, infection in humans may remain latent or lead to disease, including visceral larva migrans syndrome, ocular larva migrans syndrome, and neurotoxocarosis [44]. To reduce environmental contamination with *Toxocara* eggs (and fecal stages of other zoonotic parasites), appropriate preventive measures should be implemented in Kazakhstan. These measures include covering sandpits to protect them from contamination when not in use, regularly replacing the sand in sandpits (at least every 1–2 years) and administering anthelmintic treatment to cats on a regular basis [45].

### *Cystoisospora* spp

Infections with *C. felis* and *C. rivolta*, both non-zoonotic and of low pathogenicity in felines, are globally distributed [46], including in Russia [47–49]. However, with the exception of a local study in Kazakhstan [7], data on their occurrence in Central Asia are lacking. This study provides updated and geographically broad data on the prevalence of these species in Kazakhstan.

Oocysts of *C. felis* were detected at a higher frequency than those of *C. rivolta* (7.2% vs. 2.0%), consistent with findings in other countries [46]. Stray cats shed oocysts of both species more frequently than client-owned cats (Tables 3 and 4), in line with prior research by Dubey [46]. Shedding of *C. felis* oocysts, but not *C. rivolta*, occurred significantly more often in kittens than in adult cats (Table 3), a finding that agrees with previous observations [47, 48, 50, 51]. This can be explained by early infection and subsequent development of partial immunity.

### *T. gondii*-like coccidia

Earlier studies assessing *T. gondii*-like oocyst shedding in cats from Kazakhstan date back over five decades, reporting a prevalence of 1.6%–5.6% in Almaty [52]. The present study provides updated data, with a shedding prevalence of 0.6% in urban cats (Table 2). This figure aligns with numerous Eurasian studies, where the prevalence of shedding was generally around 1% [25, 53]. However, it can be considerably higher in specific locations; the pooled shedding prevalence in Europe and Asia has been estimated at 1.4% and 4%, respectively [54].

It is important to note that “*T. gondii*-like” oocysts may represent either zoonotic *T. gondii* or non-zoonotic *H. hammondi*. Felines are the specific definitive hosts of both coccidian species [3]. Their oocysts are morphologically indistinguishable under microscopy; differentiation is only possible through molecular diagnostics or bioassays involving mice. For instance, in a German study, *T. gondii*-like oocysts were detected in 105 of 18,259 cat feces samples, with PCR revealing 44% as *T. gondii*, 32% as *H. hammondi*, and 24% inconclusive [55]. There is no reason to assume a significantly different ratio in Kazakhstan. Therefore, the actual prevalence of *T. gondii* oocyst shedding in urban Kazakh cats may be estimated at approximately 0.4%. Future studies should incorporate molecular techniques to further clarify these data and more accurately assess zoonotic risk. Furthermore, testing soil in public areas for *T. gondii* oocysts is recommended to evaluate environmental contamination levels [56].

Toxoplasmosis remains one of the most significant parasitic zoonoses globally [6, 57]. In immunocompetent individuals, infections are usually asymptomatic or mild. However, immunocompromised patients may experience severe disease, particularly encephalitis. Pregnant women who acquire primary *T. gondii* infection during pregnancy can transmit the pathogen to the fetus, potentially resulting in miscarriage, fetopathy, or long-term sequelae such as chorioretinitis or cognitive impairment [6].

Despite the relatively low prevalence, the estimated 0.4% oocyst shedding rate represents a relevant public health concern, particularly for children who may ingest oocysts while playing in sandpits or gardens. Cats of any age can excrete millions of *T. gondii* oocysts for several consecutive days, and these oocysts are environmentally resilient, surviving for months or longer [6]. Given this, Kazakhstan’s health authorities and medical institutions should intensify public education regarding toxoplasmosis and its prevention, as has already been recommended in other countries [58].

### Giardia

In total, 6.4% of the tested cats were positive for *Giardia* coproantigen. In other countries, the prevalence of giardiosis in cats has ranged from 1% to 44% [59], with a pooled global estimate of 2.3% [60]. However, different testing methods – including microscopy, coproantigen detection, and polymerase chain reaction – have been used across studies [60]. These diagnostic methods vary considerably in sensitivity, making direct comparisons between studies challenging.

In the present study, the proportion of *Giardia*-positive samples was significantly higher in kittens up to 6 months of age than in adult cats (Table 5), which is consistent with previous findings [60, 61]. This may suggest early infection followed by gradual development of partial immunity. The higher *Giardia* positivity observed in female cats lacks a clear biological explanation and may be an incidental finding.

Giardiosis also affects humans, often with clinical symptoms [62], and such infections have been reported in Kazakhstan as well [36, 63]. Common transmission routes include waterborne, foodborne, and direct person-to-person contact [62]. Several studies have suggested that zoonotic transmission of *Giardia* may occur from pets to their owners, while others have not found supporting evidence [59, 62]. In both humans and domestic mammals, *Giardia* *duodenalis* – a species complex comprising assemblages A–H – is the primary agent of infection. Cats are mostly infected with assemblage F, which does not infect humans, but human-infective assemblages A and B have occasionally been detected in cats [59, 64, 65]. Hence, although infrequent, zoonotic transmission of *Giardia* from cats to humans remains a possibility [59, 65]. Future molecular studies are needed to identify *Giardia* genotypes circulating in Kazakh cats.

### Other parasites

*Capillaria* spp. eggs were identified in a small proportion (0.4%) of fecal samples, consistent with data from Moscow (0.5% [66]) and similar findings in other countries [20, 25]. It remains unclear whether these eggs resulted from patent infections in cats (e.g., with *Capillaria aerophila*) or were spurious due to ingestion of infected rodents or birds.

The detection of taeniid and *Dipylidium* spp. eggs in a few cats should be interpreted as incidental and does not reflect the true prevalence of cestode infection. This is because the sensitivity of fecal flotation for detecting cestodes is lower than that of methods such as helminthological necropsy and molecular techniques [3]. Previous necropsy studies have reported *Taenia taeniaeformis* in 12%–32% and *Dipylidium* spp. in 23%–40% of cats in Astana and Oral [7, 9]. The *Diphyllobothrium/Spirometra* eggs detected in one cat may belong to *Diphyllobothrium latum*, a pseudophyllid tapeworm occasionally reported in carnivores in Russia [49, 67] and Azerbaijan [68].

The absence of hookworm eggs was not unexpected, as it is consistent with negative results from cat necropsies in Kazakhstan [7, 9]. This also parallels the results of a recent study on stray dogs in Astana, which found no hookworm eggs [69]. In Moscow, 0.2% of cats tested positive for hookworm eggs [65], whereas in Vladivostok (Far Eastern Russia), the rate was 2% [49].

## CONCLUSION

This study provides the first comprehensive assessment of gastrointestinal parasites in urban cat populations across Kazakhstan. Nine distinct parasitic taxa were identified, including zoonotically significant agents such as *T. cati*, *T. gondii*-like coccidia, and *Giardia*. The overall prevalence rates of *T. cati* and *Giardia* were comparable to, or lower than, those reported in other countries; the prevalence of *T. gondii*-like oocyst shedding was low, as is typically reported.

The study’s findings underscore several practical implications. First, the shedding of *T. cati* and *T. gondii*-like oocysts by free-roaming cats highlights potential contamination of public spaces and the associated risks of human toxocarosis and toxoplasmosis. Second, these results provide essential baseline data to inform One Health strategies aimed at reducing zoonotic transmission in urban environments. Recommended measures include improving public education about the importance of these parasites, regular pet deworming, maintaining good environmental hygiene (e.g., keeping sandpits clean), and controlling stray cat populations.

Among the strengths of this study are its multicenter design, encompassing five urban centers, and the incorporation of both stray and client-owned animals, which enhances its ecological validity. However, the study’s non-probability sampling design and lack of individual animal-level metadata (e.g., deworming history, diet, housing conditions) limit the generalizability of the findings to the broader feline population.

Future research should employ molecular diagnostic techniques to distinguish *T. gondii*-like oocysts as either *T. gondii* or *H. hammondi*, determine assemblage-level genotyping of *Giardia*, and explore the spatial and seasonal variability of environmental contamination. In addition, longitudinal studies involving both cats and human populations are warranted to elucidate transmission dynamics and assess zoonotic risk more precisely.

In conclusion, this investigation establishes a foundational understanding of feline gastrointestinal parasitism in Kazakhstan’s urban settings. These findings serve as a call to action for integrated public health, veterinary, and environmental interventions aligned with the One Health framework to mitigate zoonotic threats associated with companion animals.

## AUTHORS’ CONTRIBUTIONS

LAL: Study design, supervision, and management. VK and AU: Coordination of sample collection and data curation. DMS, BEA, RSU, NEM, and IS: Sample collection and analysis. CB: Conceptualized the study, data interpretation, statistical analysis, and drafted and revised the manuscript. All authors have read and approved the final manuscript.

## References

[1] World Population Review (2024). Cat Population by Country 2024.

[2] TAÑBA in Digits (2025). Animal Accounting Information System.

[3] Deplazes P, Eckert J, Mathis A, Von Samson-Himmelstjerna G, Zahner H (2016). Parasitology in Veterinary Medicine.

[4] Foreman-Worsley R, Finka L.R, Ward S.J, Farnworth M.J (2021). Indoors or outdoors?An international exploration of owner demographics and decision making associated with lifestyle of pet cats. Animals (Basel).

[5] Maciag L, Morgan E.R, Holland C (2022). *Toxocara*:Time to let cati 'out of the bag'. Trends Parasitol.

[6] Dubey J.P (2022). Toxoplasmosis of Animals and Humans.

[7] Lider L.A, Akibekov O.S, Tokpanov S.S, Ibraev N.E, Borovikov S.N (2010). Helminths of domestic carnivores in the Akmola region. Bull. Sci. Saken Seifullin Kazakh Agrotech. Univ.

[8] Sultanov A, Abdybekova A, Abdibaeva A, Shapiyeva Z, Yeshmuratov T, Torgerson P.R (2014). Epidemiology of fishborne trematodiasis in Kazakhstan. Acta Trop.

[9] Aboimova A.P, Karmaliev R.S (2016). Helminths of domestic cat (*Felis domestica*) in the city of Uralsk. Theory Pract. Parasit. Dis. Control 2016.

[10] Karmaliyev R, Nurzhanova F, Sidikhov B, Murzabaev K, Sariyev N, Satybayev B, Abirova I (2023). Epizootiology of opisthorchiasis in carnivores, fish and mollusks in the West Kazakhstan Region. Am. J. Anim. Vet. Sci.

[11] Sidikhov M.B, Yertleuova O.B, Gabdullin D.Y, Dushayeva Z.L (2023). The prevalence of invasive diseases among cats in Uralsk. Bull. Sci. Saken Seifullin Agrotechn. Res. Univ. Vet. Sci.

[12] Lider L, Ussenbayev A, Kiyan V, Kurenkeyeva D, Seitkamzina D, Akmambayeva B, Uakhit R, Smagulova A, Sytni I (2024). Prevalence of *Giardia duodenalis* in household and shelter cats in Almaty, South-Eastern Kazakhstan. Am. J. Anim. Vet. Sci.

[13] Geodatos (2025). Geographic Coordinates Finder. https://www.geodatos.net/en/coordinates.

[14] Bureau of National Statistics (2025). Agency for Strategic Planning and Reforms of the Republic of Kazakhstan. Demographic Statistics.

[15] Beck H.E, Zimmermann N.E, McVicar T.R, Vergopolan N, Berg A, Wood E.F (2018). Present and future Köppen-Geiger climate classification maps at 1-km resolution. Sci. Data.

[16] Mangan A, Lawlor A, De Waal T, Aungier S (2019). The prevalence of intestinal parasites in dogs and cats in the greater Dublin area. Vet. Irel. J.

[17] MEGACOR (2020). Data sheet FASTest®CRYPTO-GIARDIA strip comparative study 2012-2020. Megacor Company, Hörbranz, Austria.

[18] Ackermann H (2010). BIAS für Windows. Ver. 9.05. Epsilon, Hochheim.

[19] Thrusfield M (1997). Veterinary Epidemiology.

[20] Beugnet F, Bourdeau P, Chalvet-Monfray K, Cozma V, Farkas R, Guillot J, Halos L, Joachim A, Losson B, Miró G, Otranto D, Renaud M, Rinaldi L (2014). Parasites of domestic owned cats in Europe:Co-infestations and risk factors. Parasit. Vectors.

[21] Yang Y, Liang H (2015). Prevalence and risk factors of intestinal parasites in cats from China. Biomed. Res. Int.

[22] Lucio-Forster A, Mizhquiri Barbecho J.S, Mohammed H.O, Kornreich B.G, Bowman D.D (2016). Comparison of the prevalence of *Toxocara* egg shedding by pet cats and dogs in the U.S.A., 2011–2014. Vet. Parasitol. Reg. Stud. Rep.

[23] Moskvina T.V, Izrailskaia A.V, Tsybulsky A.V (2018). Parasites of stray and client-owned domestic cats in urban areas in Russia during 2000–2015 years. Trop. Biomed.

[24] Sarvi S, Daryani A, Sharif M, Rahimi M.T, Kohansal M.H, Mirshafiee S, Siyadatpanah A, Hosseini S, Gholami S (2018). Zoonotic intestinal parasites of carnivores:A systematic review in Iran. Vet. World.

[25] Globokar Vrhovec M, Alnassan A.A, Pantchev N, Bauer C (2022). Is there any change in the prevalence of intestinal or cardiopulmonary parasite infections in companion animals (dogs and cats) in Germany between 2004–2006 2015–2017?An assessment of the impact of the first ESCCAP guidelines. Vet. Parasitol.

[26] Kurnosova O.P, Panova O.A, Arisov M.V (2023). The prevalence of potentially zoonotic intestinal parasites in dogs and cats in Moscow, Russia. Helminthologia.

[27] Rostami A, Sepidarkish M, Ma G, Wang T, Ebrahimi M, Fakhri Y, Mirjalali H, Hofmann A, Macpherson C.N.L, Hotez P.J, Gasser R.B (2020). Global prevalence of *Toxocara* infection in cats. Adv. Parasitol.

[28] Bonilla-Aldana J.L, Espinosa-Nuñez A.C, Bonilla-Aldana D.K, Rodriguez-Morales A.J (2024). *Toxocara cati* infection in cats (*Felis catus*):A systematic review and meta-analysis. Animals (*Basel*),.

[29] Sprent J.F.A (1956). The life history and development of *Toxocara cati* (Schrank 1788) in the domestic cat. Parasitology.

[30] Swerczek T.W, Nielsen S.W, Helmboldt C.F (1971). Transmammary passage of *Toxocara cati* in the cat. Am. J. Vet. Res.

[31] Coati N, Schnieder T, Epe C (2004). Vertical transmission of *Toxocara cati* Schrank 1788 (*Anisakidae*) in the cat. Parasitol. Res.

[32] Ma G, Rostami A, Wang T, Hofmann A, Hotez P.J, Gasser R.B (2020). Global and regional seroprevalence estimates for human toxocariasis:A call for action. Adv. Parasitol.

[33] Moskvina T.V, Bartkova A.D, Ermolenko A.V (2016). Geohelminths eggs contamination of sandpits in Vladivostok, Russia. Asian Pac. J. Trop. Med.

[34] Keegan J.D, Airs P.M, Brown C, Dingley A.R, Courtney C, Morgan E.R, Holland C.V (2025). Park entrances, commonly contaminated with infective *Toxocara canis* eggs, present a risk of zoonotic infection and an opportunity for focused intervention. PLoS Negl. Trop. Dis.

[35] Akhmadishina L.V, Ruzina M.N, Lukasheva M.A, Kyuregyan K.K, Mikhailov M.I, Lukasheva A.N (2020). Seroprevalence and incidence of human toxocarosis in Russia. Adv. Parasitol.

[36] Proskurina L, Koltun G, Simakova M, Repsh N, Belov A (2020). Prevalence of opisthorchiasis in the Pavlodar region of the Republic of Kazakhstan. E3S Web Conf.

[37] Shabdarbayeva G, Yalysheva S (2020). A retrospective analysis of the prevalence of echinococcosis in the Republic of Kazakhstan. Bull. Nat. Acad. Sci. Rep. Kazakhstan 2020.

[38] Kirpicheva U, Shapiyeva Z (2023). Parasitic diseases surveillance in Kazakhstan:Incidence trends and future projections. Open Forum Infect. Dis.

[39] Torgerson P.R, Rosenheim K, Tanner I, Ziadinov I, Grimm F, Brunner M, Shaiken S, Shaikenov B, Rysmukhambetova A, Deplazes P (2009). Echinococcosis, toxocarosis and toxoplasmosis screening in a rural community in eastern Kazakhstan. Trop. Med. Int. Health.

[40] Swerczek T.W, Nielsen S.W, Helmboldt C.F (1970). Ascariasis causing pulmonary arterial hyperplasia in cats. Res. Vet. Sci.

[41] Dillon A.R, Tillson D.M, Hathcock J, Brawner B, Wooldridge A, Cattley R, Welles B, Barney S, Lee-Fowler T, Botzman L, Sermersheim M, Garbarino R (2013). Lung histopathology, radiography, high-resolution computed tomography, and bronchio-alveolar lavage cytology are altered by *Toxocara cati* infection in cats and is independent of development of adult intestinal parasites. Vet. Parasitol.

[42] Janecek E, Waindok P, Bankstahl M, Strube C (2017). Abnormal neurobehaviour and impaired memory function as a consequence of *Toxocara canis*- as well as *Toxocara cati*-induced neurotoxocarosis. PLoS Negl. Trop. Dis.

[43] Poulsen C.S, Yoshida A, Wellbrant T.T, Leifsson P.S, Skallerup P, Thamsborg S.M, Nejsum P (2024). Migratory pattern of zoonotic *Toxocara cati* and *T. canis* in experimentally infected pigs. Eur. J. Clin. Microbiol. Inf. Dis.

[44] Auer H, Walochnik J (2020). Toxocariasis and the clinical spectrum. Adv. Parasitol.

[45] ESCCAP (2021). ESCCAP Guideline 01. Worm Control in Dogs and Cats. European Scientific Council Companion Animal Parasites Secretariat.

[46] Dubey J.P (2020). Coccidiosis in cats (*Felis catus*). In:Dubey, J.P., editor. Coccidiosis in Livestock, Poultry, Companion Animals, and Humans. CRC Press, Boca-Raton.

[47] Moskvina T, Zheleznova L (2017). Parasitic diseases of dogs and cats in the city of Vladivostok. Russ. J. Parasitol.

[48] Pachetnik V.E (2024). Protozoonosis and most important helminthozoonosis in domestic cats in Moscow. Theory Pract. Parasit. Dis. Control.

[49] Tabakaeva T.V, Galkina I.V, Tabakaev A.V, Shchelkanov MY (2024). Anthropozoonotic parasitoses of dogs and cats in the urban ecosystem of Vladivostok, Russia. S. Russ. Ecol. Dev.

[50] Gates M.C, Nolan T.J (2009). Endoparasite prevalence and recurrence across different age groups of dogs and cats. Vet. Parasitol.

[51] Barutzki D, Schaper R (2011). Results of parasitological examinations of faecal samples from cats and dogs in Germany between 2003 2010. Parasitol. Res.

[52] Beyer T.V, Shevkunova E.A (1986). A review of toxoplasmosis of animals in the U.S.S.R. Vet. Parasitol.

[53] Dubey J.P, Cerqueira-Cézar C.K, Murata F.H.A, Kwok O.C.H, Yang Y.R, Su C (2020). All about toxoplasmosis in cats:The last decade. Vet. Parasitol.

[54] HatamNahavandi K, CaleroBernal R, Rahimi M.T, Pagheh A.S, Zarean M, Dezhkam A, Ahmadpour E (2021). *Toxoplasma gondii* infection in domestic and wild felids as public health concerns:A systematic review and metaanalysis. Sci. Rep.

[55] Herrmann D.C, Pantchev N, Globokar Vrhovec M, Barutzki D, Wilking H, Fröhlich A, Lüder C.G.K, Conraths F.J, Schares G (2010). Atypical *Toxoplasma gondii* genotypes identified in oocysts shed by cats in Germany. Int. J. Parasitol.

[56] Maleki B, Ahmadi N, Olfatifar M, Gorgipour M, Taghipour A, Abdoli A, Khorshidi A, Foroutan M, Mirzapour A (2021). *Toxoplasma* oocysts in the soil of public places worldwide:A systematic review and meta-analysis. Trans. R. Soc. Trop. Med. Hyg.

[57] Rostami A, Riahi S.M, Gamble H.R, Fakhri Y, Shiadeh M.N, Danesh M, Behniafar H, Paktinat S, Foroutan M, Mokdad A.H, Hotez P.J, Gasser R.B (2020). Global prevalence of latent toxoplasmosis in pregnant women:A systematic review and meta-analysis. Clin. Microbiol. Inf.

[58] Smith N.C, Goulart C, Hayward J.A, Kupz A, Miller C.M, Van Dooren G.G (2021). Control of human toxoplasmosis. Int. J. Parasitol.

[59] Barbosa A.D, Egan S, Feng Y, Xiao L, Ryan U (2023). *Cryptosporidium* and *Giardia* in cats and dogs:What is the real zoonotic risk?. Curr. Res. Parasitol. Vector Borne Dis.

[60] Bouzid M, Halai K, Jeffreys D, Hunter P.R (2015). The prevalence of *Giardia* infection in dogs and cats, a systematic review and meta-analysis of prevalence studies from stool samples. Vet. Parasitol.

[61] Kurnosova O.P, Panova O.A, Arisov M.V (2024). Prevalence of *Giardia duodenalis* in dogs and cats:Age-related predisposition, symptomatic, and asymptomatic cyst shedding. Vet. World.

[62] Krumrie S, Capewell P, Smith-Palmer A, Mellor D, Weir W, Alexander C.L (2022). A scoping review of risk factors and transmission routes associated with human giardiasis outbreaks in high-income settings. Curr. Res. Parasitol. Vector Borne Dis.

[63] Begaydorova R, Nasakaeva G, Tabagari S, Yukhnevich Y, Alshynbekova G (2014). Clinical and diagnostic features and treatment of giardiasis. Georgian Med. News.

[64] Ramírez-Ocampo S, Cotte-Alzate J.D, Escobedo A.A, Rodríguez-Morales A.J (2017). Prevalence of zoonotic and non-zoonotic genotypes of *Giardia intestinalis* in cats:A systematic review and meta-analysis. Infez. Med.

[65] Cai W, Ryan U, Xiao L, Feng Y (2021). Zoonotic giardiasis:An update. Parasitol. Res.

[66] Kurnosova O.P, Arisov M.V, Adoyevskaya,xI M (2019). Intestinal parasites of pets and other house-kept animals in Moscow. Helminthologia.

[67] Moskvina T.V, Ermolenko A.V (2016). Helminth infections in domestic dogs from Russia. Vet. World.

[68] Fataliev G.G, Ibragimova R.S (2016). Comparative analysis of the helminth fauna in cats of the family Felidae in Azerbaijan. Russ. J. Parasitol.

[69] Bauer C, Lider L.A, Ussenbayev A.E, Seitkamzina D.M, Zhanabayev A.A, Maksimov P, Knaus M (2024). *Toxascaris leonina* in dogs - a nematode species of high prevalence in some regions of Eurasia. Vet. Parasitol. Reg. Stud. Rep.

